# Native ambient mass spectrometry of membrane proteins directly from bacterial colonies†

**DOI:** 10.1039/d4cc03881a

**Published:** 2025-02-05

**Authors:** Yuying Du, Helen J. Cooper

**Affiliations:** a School of Biosciences, University of Birmingham, Edgbaston Birmingham B15 2TT UK h.j.cooper@bham.ac.uk

## Abstract

Native ambient mass spectrometry (NAMS) enables analysis of protein structure directly from biological substrates by use of liquid junction sampling techniques together with sampling solvents which mimic the proteins’ natural environment. Here, we demonstrate detection of membrane and membrane-associated proteins directly from *E. coli* by combining liquid extraction surface analysis (LESA) with a straightforward washing protocol, which attenuates soluble proteins and enables detection of membrane proteins.

## Abstract 2

Liquid extraction surface analysis mass spectrometry of membrane proteins directly from living bacterial colonies.

## Graphical abstract

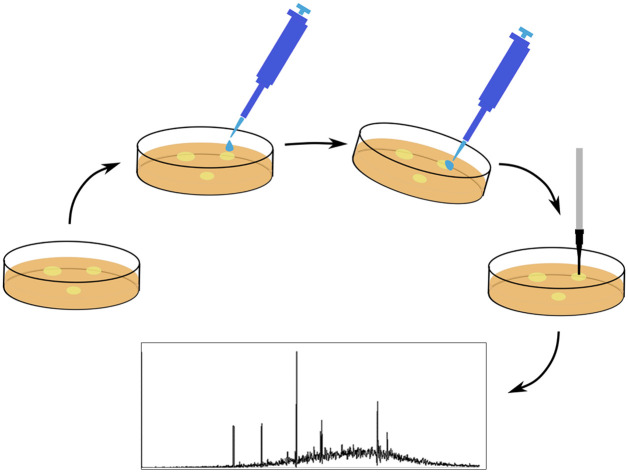

Native ambient mass spectrometry (NAMS)^1,2^ combines native mass spectrometry^3–5^ with ambient mass spectrometry^6^ to directly analyse biological substrates such as bacterial colonies^7^ and thin tissue sections^2^ without complicated sample preparation, while maintaining solution-phase noncovalent interactions into the gas phase. We have previously demonstrated NAMS of proteins and their complexes from living colonies of *Escherichia coli* K12 (*E. coli* K12) by use of liquid extraction surface analysis (LESA) mass spectrometry;^7^ however, to date, all of those detected have been soluble proteins. In parallel work, we have shown NAMS detection of membrane proteins in mammalian tissue sections by tailoring the extraction solvent^8^ and use of tissue washing.^1^

Membrane proteins play a crucial role in essential cellular functions, such as signal transduction, apoptosis, and metabolism.^9,10^ They constitute 50% of all known drug targets.^11^ Understanding of the structures of membrane proteins is therefore important for unravelling their biochemical mechanisms and advancing the development of novel therapeutics. Due to the naturally low abundance of membrane proteins and the highly heterogeneous and insoluble nature of the membrane environment, characterization of membrane protein structure, function, and interactions poses a significant challenge.^9^ One of the most commonly-applied methods to address this is to use MS-compatible detergents – amphipathic molecules that can solubilise membrane proteins while preserving the non-covalent interactions for native MS analysis.^12–14^

Additional challenges for analysis of membrane proteins in Gram-negative bacteria are posed by the structure of the cell wall, which comprises an inner membrane, a peptidoglycan layer and the outer membrane.^15^ To address these challenges presented by the hydrophobic nature and low abundance of membrane proteins, together with the challenge of the inherent complexity presented by direct sampling of bacterial colonies, *i.e.*, the presence of soluble proteins and other molecules which can complicate or even attenuate detection of membrane proteins, we designed and optimised a workflow which incorporates washing steps and NAMS for directly detecting membrane proteins from living *E. coli* colonies.

We have shown previously that washing is beneficial for detection of membrane proteins from mammalian tissue by native ambient mass spectrometry.^1^ Here, we employed a washing solution consisting of 200 mM ammonium acetate containing 0.5× the critical micelle concentration (CMC; 0.125% v/v) of the detergent octyl tetraethylene glycol ether (C8E4). (A range of washing solvents were trialled including 200 mM ammonium acetate (without C8E4), 200 mM ammonium acetate with 0.5 × CMC C8E4, and 200 mM ammonium acetate with 2 × CMC C8E4, see Fig. S1, ESI†). C8E4 is a non-ionic and comparatively mild MS-compatible detergent which is suitable for transfer of membrane proteins into the gas phase without affecting protein–protein interactions.^12,16^ For the washing steps, we employed a concentration of C8E4 below its CMC to disrupt protein–lipid interactions without reconstituting membrane proteins into detergent micelles.^12^

Fig. S2 (ESI†) illustrates the workflow employed. Bacterial colonies were cultured on agar plates. Two washing protocols were trialled. In the first, the bacteria were washed three times followed by a wash with 200 mM ammonium acetate (without detergent), making a total of four initial washes which were then repeated (denoted here as 2 × (3 + 1)). In the second washing protocol, the bacteria were washed three times followed by a final wash with 200 mM ammonium acetate (without detergent) (denoted here as (3 + 1)). After washing, the colonies were sampled by liquid extraction surface analysis (LESA) and the extracts were transferred into home-made gold-coated borosilicate tips and electrosprayed into the mass spectrometer. A total of 10 proteins were identified in this work and are summarised in Tables S1 and S2 (ESI†). Although there is known variability in LESA sampling efficiency, the proteins identified were detected in all replicates following the respective washing protocols.

Fig. 1A shows representative mass spectra obtained following the 2 × (3 + 1) washing protocol. Peaks corresponding to two membrane proteins, outer membrane protein A (OmpA) and long-chain fatty acid transport protein (FADL) were observed. The proteins were identified by higher-energy collision dissociation (HCD), see Fig. 2. Full details of protein identifications are given in the ESI.† Sequence coverages of 3% (FADL) and 2% (OMPA) were obtained; however, all cleavages observed were either C-terminal to aspartic acid or N-terminal to proline. These cleavage sites are known to be favoured^17^ in native mass spectrometry and provide additional confidence in the protein assignment. Both of these proteins are outer membrane proteins. FADL is responsible for cellular transport of long-chain fatty acids^18,19^ and their conversion to acyl-CoA.^18^ OMPA serves several important functions including maintaining the stability and integrity of the outer membrane,^20–22^ anchoring it to the peptidoglycan layer,^21,22^ contributing to the permeability barrier^23^ and regulating the passage of molecules, including ions and nutrients.^21^ OmpA was detected with 2 Da mass shift, indicating the presence of a disulfide bond between C290 and C302.

**Fig. 1:**
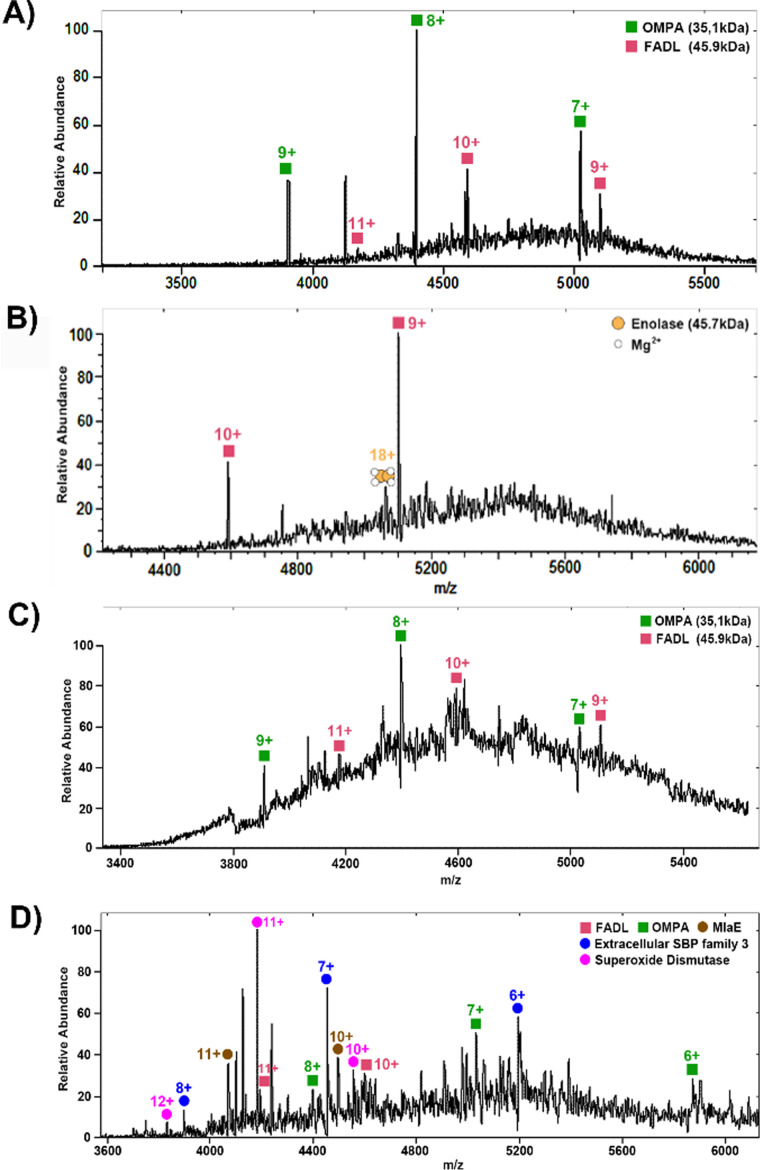
Representative full-scan mass spectrum obtained following eight rounds of washing (A) and (B) and four rounds (C) and (D) of washing. (A) Mass spectrum with compensation scaling factor 5%. (B) Mass spectrum with CSF 7%. (C) Mass spectrum with CSF 5%. (D) Mass spectrum with CSF 7%.

**Fig. 2:**
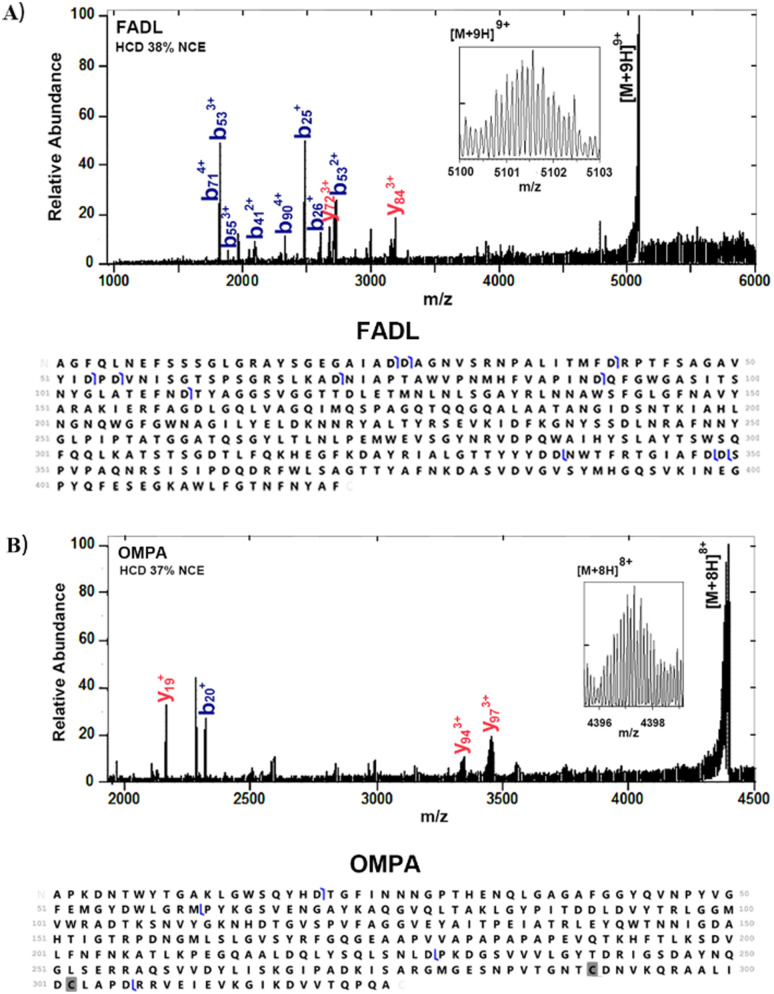
Identification of FADL and OMPA following the 2 × (3 + 1) washing protocol. (A) HCD mass spectrum of 9+ ions (see inset) of FADL (*m*/*z* 5102 ± 5), NCE38%, and sequence coverage. (B) HCD mass spectrum of 8+ ions (see inset) of OMPA (*m*/*z* 4397 ± 5), NCE 37%, and sequence coverage.

The initial washing method resulted in the detection of just two membrane proteins. To improve transmission of higher mass ions, the compensation scaling factor was increased from 5% to 7%, Fig. 1B. Under these conditions, a further protein was detected and identified as the soluble enolase dimer but no further membrane proteins were detected. Presumably, the removal of smaller, higher solubility proteins during the colony washing process enabled the detection of this soluble protein. Enolase is a magnesium-binding protein and the mass difference between the calculated and measured masses can be ascribed in part to the binding of two Mg^2+^ ions to each enolase subunit.

The detection of just two membrane proteins suggests that the protocol may be too harsh. In subsequent experiments, the number of washing steps was reduced. Fig. 1C and D show representative mass spectra obtained following the (3 + 1) washing protocol. Under these washing conditions and with a compensation scaling factor of 5%, the membrane proteins OMPA and FADL were again detected. At a compensation scaling factor of 7%, several soluble proteins, all of which were cytoplasmic proteins, were identified (see Table S2, ESI†). Several of these were observed with considerable mass shifts between the measured and calculated masses: superoxide dismutase is a Mn-binding protein and the mass shift can be attributed to binding of one Mn^2+^ ion to each subunit. Phosphoenolpyruvate carboxykinase and serine hydroxymethyl transferase exhibited differences of 324 Da and 578 Da respectively between their calculated and measured masses, suggesting the presence of either ligand binding or large modification. The two membrane proteins FADL and OMPA were also observed, but did not show good reproducibility. In addition, three membrane-related proteins were identified: extracellular solute-binding protein (SBP) family 3, maltodextrin-binding protein (MalE) and cationic amino acid ABC transporter (CAT)-periplasmic binding protein (see Fig. S3, ESI†). Full details of protein identifications are given in the ESI.† For SBP-family 3 and MalE, it was possible to determine the accurate mass directly as high resolution mass spectrometry provided isotopic resolution. For CAT-PBP, however, the low signal-to-noise precluded isotopic resolution and it was necessary to employ proton transfer charge reduction MS to determine the intact mass of the protein. PTCR MS^24,25^ involves an ion–ion reaction between multiply-charged protein ions and the PTCR reagent anion resulting in proton transfer. Deconvolution of the resulting charge-reduced product ion spectrum yields the intact mass.

Bacterial extracellular SBPs have multiple roles in cellular functions,^26^ including acting as chemoreceptors, recognizing specific molecules, serving as key components in transport systems,^26,27^ and initiating signal transduction pathways to trigger cellular responses.^26,28^ MalE is a periplasmic binding protein and a part of the ATP-binding cassette (ABC) transporter complex MalEFGK that works with maltodextrin permease to transport maltodextrins into the bacterial cell.^29,30^ The CAT-periplasmic binding protein is a part of CAT ABC transporter system that helps bring cationic amino acids into the bacterial cell.^31,32^ Overall, decreasing the number of washing steps resulted in lower signal-to-noise ratios for the membrane protein peaks and lower sequence coverage following MS2 fragmentation (see Tables S1 and S2, ESI†). More soluble proteins were observed with fewer wash steps; however, the larger soluble protein dimer enolase was only observed and identified with the 2 × (3 + 1) wash (see Table S1, ESI†). We hypothesise that the harsher washing protocol removed the overlapping serine hydroxymethyltrasferase, but not the enolase, enabling its detection. Interestingly, this strain of *E. coli* is engineered to overexpress the membrane protein ZipA and this was not observed following either of the washing protocols. One potential explanation may stem from the structural features of the bacteria, which commonly possesses two membranes. Although all the membrane-related proteins detected are periplasmic proteins associated with inner membrane proteins, all the membrane proteins observed here are outer membrane. ZipA, however, is an inner membrane protein.

In conclusion, our findings demonstrate that colony washing prior to LESA extraction and mass spectrometry analysis effectively diminishes or eliminates signals of soluble proteins and spectrum noise. This process facilitates the direct detection of integral membrane and membrane-associated proteins within living bacterial colonies. Notably, the signal-to-noise ratio for outer membrane proteins improved post-washing. Subsequent investigations will focus on exploring membrane proteins in different bacterial strains, yeast, and applications of alternative detergents, with the goal of enhancing the detection of membrane proteins across various microorganisms.

YD was funded by the Darwin Trust. HJC was funded by EPSRC (EP/S002979/1). The Orbitrap Eclipse mass spectrometer used in this work was funded by BBSRC (BB/S019456/1).

## Data availability

Data for this article, including mass spectrometry data and MS/MS data are available at the University of Birmingham Institutional Research Archive at https://doi.org/10.25500/edata.bham.00001240.

## Conflicts of interest

There are no conflicts to declare.

## Supplementary Material

CC-061-D4CC03881A-s001
