# Supplementary material for: Native ambient mass spectrometry of membrane proteins directly from bacterial colonies
Source: Chem Commun (Camb). 2025 Feb 5;61(21):4168–71. doi: 10.1039/d4cc03881a (PMC11833770; doi:10.1039/d4cc03881a)
Supplement: CC-061-D4CC03881A-s001 [file CC-061-D4CC03881A-s001.pdf]

Supplementary Information (SI) for ChemComm.
This journal is © The Royal Society of Chemistry 2025

Supporting Information

for

Native ambient mass spectrometry of membrane proteins directly from bacterial colonies

Yuying Du and Helen J. Cooper
School of Biosciences, University of Birmingham, Edgbaston, Birmingham B15 2TT, UK.

Contents

Experimental methods

Figure S1. Replicates of protocols based on different washing solvents.

Figure S2. Schematic of workflow

Section 1

Table S1: Protein identification with washing protocol 2x(3+1)

Protein identification information of washing protocol 2x(3+1)

Section 2

Table S2: Protein identification with washing protocol 3+1

Figure S3. Identification of membrane associated proteins

Protein identification information of washing protocol 3+1

References

Experimental

Material

Lysogeny broth (LB) consisting of 5g/L yeast extract (VWR, Lutterworth, UK), 10 g/L peptone (Sigma-Aldrich, Gillingham, UK), and 10 g/L sodium chloride (Fisher Scientific, Loughborough, UK) for culturing the *Escherichia coli* BL21 (DE3) strain was prepared. Lysogeny broth agar (LBA) was prepared by dissolving 20 g/L of pre-mixed LB broth base and 15 g/L of agar (VWR International, Leuven, Belgium) in purified (18 MΩ) water, followed by sterilisation by autoclaving at 121°C and 15-16 psig. The stock of *Escherichia coli* BL21 (DE3) overexpressing ZipA was provided by Professor Timothy Dafforn, University of Birmingham, UK. HPLC grade ammonium acetate was purchased from J. T. Baker (Deventer, Netherlands), while the MS-grade water was obtained from Fisher Scientific (Loughborough, UK). The detergent C8E4 was purchased from Sigma-Aldrich (Gillingham, UK).

Sample preparation

Colonies of *Escherichia coli* BL21 (DE3) were raised in 5−10 mL of liquid broth. After incubating at 37 °C overnight, samples were taken by a 5µL inoculating loop which was then inoculated overnight on solid LBA medium in 60 mm diameter Petri dishes.

Washing of bacterial colonies

Twenty µL of the washing solvent (200mM ammonium acetate with 0.5 CMC C8E4) was pipetted onto the colony, ensuring the colony was completely covered. After 30 seconds, the washing solvent was gently aspirated whilst tipping the plate to allow collection of the washing droplet at the edge of the colony. After three rounds of washing with this solvent, a wash with 200mM ammonium acetate was performed. This washing protocol was performed either once or twice, for a total of four (denoted (3+1) herein) or eight (denoted 2x(3+1)) washing steps, as described in the main text.

LESA mass spectrometry

LESA was performed by use of a TriVersa NanoMate (Advion, Ithaca, NY). The TriVersa Nanomate was controlled by the advanced user interface (AUI) feature of the TriVersa NanoMate ChipSoft Manager software 8.3.3. A total of 3 µL of the solvent comprising 200 mM ammonium acetate with 2x CMC C8E4 was aspirated from the solvent well and the pipette was brought into contact with the colony surface (contact LESA[1]). Two µL of solvent was dispensed onto the colony. After maintaining the liquid junction for 3s, the solvent was then aspirated. This dispense/reaspiration cycle was repeated 10 times. The sample was placed into a well in a 96-well plate placed within the TriVersa Nanomate and subsequently transferred to a gold-coated borosilicate nanoelectrospray emitter for introduction into the mass spectrometer.

The Orbitrap Eclipse mass spectrometer (Thermo Fisher Scientific, San Jose) was operated in positive, intact protein mode. For the samples obtained following eight rounds of washing (i.e., the 2x(3+1) washing protocol), the mass spectrometer was operated in ion routing

multipole (IRM) high-pressure mode (20 mTorr) with in-source collision energy of 130 V. Full scan mass spectra were acquired in the range m/z 2000-8000 at a resolving power of 7,500 at m/z 200, with compensation scaling of either 5% and 7%.

For samples obtained following four rounds of washing (i.e., the (3+1) washing protocol), the mass spectrometer was operated in high-pressure mode with an in-source collision energy of 110 V. Mass spectra were acquired at a resolving power of 7,500 at m/z 200, with compensation scaling of either 5% and 7%. To aid desolvation, a two-step wide isolation window was applied (m/z 5000 ± 2000) with 30% normalised collision energy (NCE) HCD applied in the first step and 5% NCE HCD in the second step. For this HCD clean-up, NCE was normalised to 10+ charges.

Protein identification was achieved by HCD MS/MS (with or without prior HCD clean-up). In HCD experiments for proteins <32 kDa, the instrument was operated in standard pressure mode. In all other HCD experiments, the instrument was operated in high pressure mode. Precursor ions were isolated with an isolation window of 15-20 and subjected to HCD between 20%-50% NCE. The fragments were detected in the orbitrap analyser at a resolution of 120,000 at m/z 200 (standard pressure mode) or 7500 at m/z 200 (high pressure mode).

PTCR experiments were performed at a resolution of 7500 at m/z 200 with a reaction time between 2-10ms dependent on each protein.

Protein identification.

Top-down protein identification was achieved by use of ProSightPC software, version 4.1 alpha (Thermo Fisher Scientific, Bremen, Germany). The whole organism proteome database for Escherichia coli BL21 (DE3) (UP000002032, 4156 entries) was downloaded in XML format from the UniProt Web site (uniprot.org). The database was structured as a standard top-down database, accounting for initial methionine cleavage, N-terminal acetylation, and N-terminal formylation, while also incorporating single-nucleotide polymorphisms (SNPs) and post-translational modifications (PTMs). The MS/MS spectra raw data were saved using Freestyle (Thermo Fisher Scientific). Deconvolution used the THRASH algorithm [2] within the ProSightPC import profile window, employing default parameters and an S/N ratio ranging from 0.5 to 2, depending on the quality of the acquired MS/MS spectra. Absolute mass search incorporated a delta-mass (Δm) option to identify potential unknown post-translational modifications and mutations. The search window width was set to 1000 Da with a fragment tolerance of ±20 ppm, and a minimum of 2 matching fragments was required. Identified protein sequences were validated using the Sequence Gazer function in ProSightPC software, followed by manual peak assignment. Protein identities were confirmed by manual analysis using Protein Prospector (http://prospector.ucsf.edu/prospector/mshome. htm).

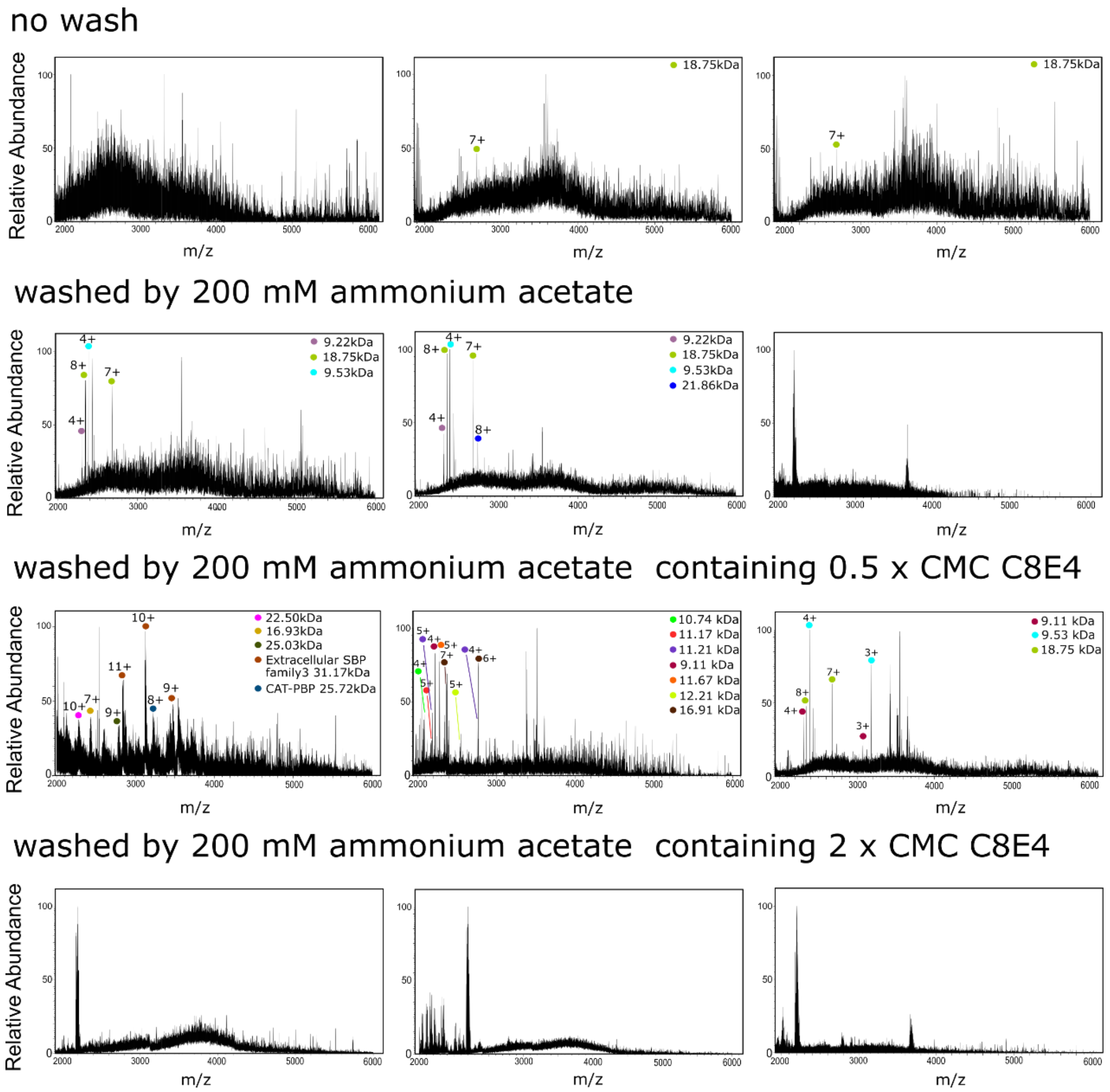

Figure S1. Optimisation of washing solvent: LESA mass spectra obtained following washing (single round) of *E. coli* colonies with solvents comprising 200 mM ammonium acetate and various concentrations of C8E4. Experiments were performed in triplicate.

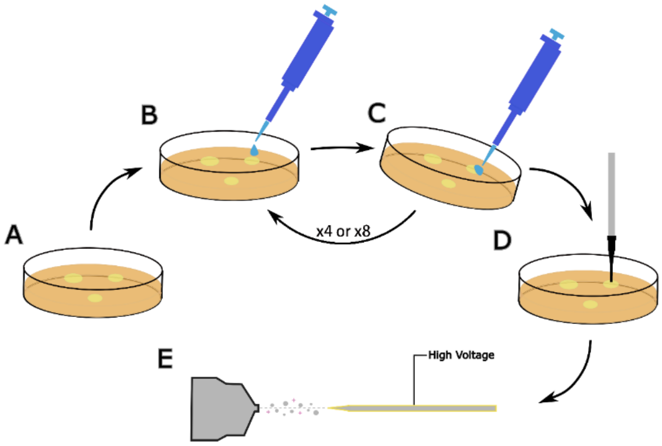

Figure S2. Schematic of workflow. A) Bacteria were cultured on agar and grew as single colonies. **B)** Washing solvent comprising 200mM ammonium acetate with or without 0.5x CMC C8E4 (0.125% v/v) was deposited on the colony. **C)** The plate was gently tilted and the washing solvent aspirated and sent to waste. **D)** The colony was sampled by LESA. Extraction solvent comprised 200 mM ammonium acetate containing 2x CMC C8E4 (0.5% v/v). **E)** The sample was transferred to a home-made gold coated borosilicate tip and introduced to the mass spectrometer by electrospray ionisation.

Section 1: Identification of proteins detected following washing protocol 2x(3+1)

Table S1: Summary of proteins identified

Washing Protocol	Protein	m/z	Charge state	Protein mass calc (Da)	Protein mass observed (Da)	Mass difference (Da)	Sequence Coverage	Protein Stoichiometry Observed	Protein Type
2x(3+1)	Long-chain fatty acid transport protein	5102	9+	45878.1115	45878.0624	-0.0491	3%	monomer	membrane protein
2x(3+1)	Outer membrane protein A	4397	8+	35150.5051	35148.4338	-2.0713	2%	monomer	membrane protein
2x(3+1)	Enolase	5085	18+	90990.7780 (dimer)	91102.0000(dimer)	111.222(dimer)	2%	homodimer	soluble protein

Details of protein identification following washing protocol 2x(3+1)

1. Protein name: Long-chain fatty acid transport protein (FADL)

Charge state: +9

Observed monoisotopic mass: 45878.0624 Da

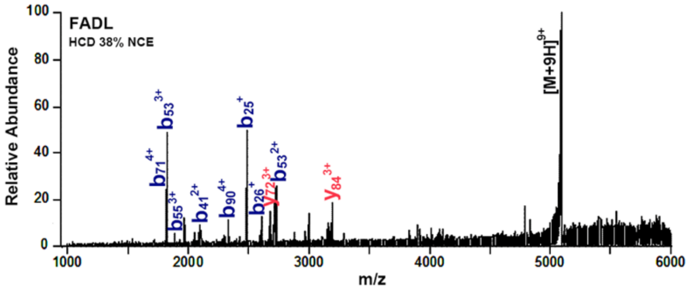

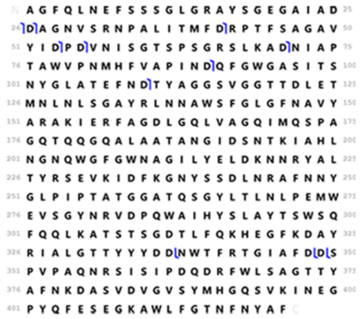

Name	m/z Monoisotopic	Mass Monoisotopic	Theoretical Mass	Error (Da)	Error (ppm)
b25	2486.1531	2485.1459	2485.1509	-0.0051	-2.0389
b26	2601.1772	2600.1700	2600.1779	-0.0079	-3.0436
b41	2094.4803	4186.9460	4186.9654	-0.0193	-4.6131
b53	2733.2987	5464.5829	5464.6058	-0.0229	-4.1880
b53	1822.5378	5464.5917	5464.6058	-0.0141	-2.5780
b55	1893.2233	5676.6480	5676.6855	-0.0375	-6.6000
b71	1812.6216	7246.4574	7246.4965	-0.0392	-5.4061
b90	2334.6295	9334.4890	9334.5216	-0.0326	-3.4973
b110	2874.3621	11493.4192	11493.4812	-0.0620	-5.3949
y72	2679.6019	8035.7838	8035.8281	-0.0442	-5.5029
y72	4018.9062	8035.7979	8035.8281	-0.0302	-3.7529
y73	2717.9450	8150.8131	8150.8550	-0.0419	-5.1404
y73	4076.4141	8150.8137	8150.8550	-0.0413	-5.0663
y84	3154.1545	9459.4417	9459.4801	-0.0384	-4.0636

2. Protein name: Outer membrane protein A (OMPA)

Charge state: +8

Observed monoisotopic mass: 35148.4338 Da

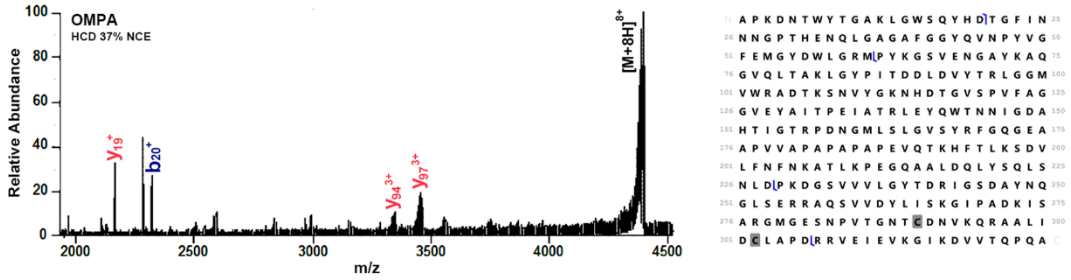

Name	m/z Monoisotopic	Mass Monoisotopic	Theoretical Mass	Error (Da)	Error (ppm)
b20	2320.0690	2319.0617	2319.0709	-0.0091	-3.9395
y19	2165.2337	2164.2265	2164.2328	-0.0063	-2.9050
y94	3347.0385	10038.0937	10038.1541	-0.0604	-6.0182
y97	3460.4298	10378.2675	10378.3287	-0.0612	-5.8956
y324	4385.6516	35077.1547	35077.4445	-0.2898	-8.2611

3.Protein name: Enolase

Charge state: +13, +18

Observed monoisotopic mass: 45551 Da, 91102 Da (Dimer) (with 2 Mg^{2+} ions each subunit)

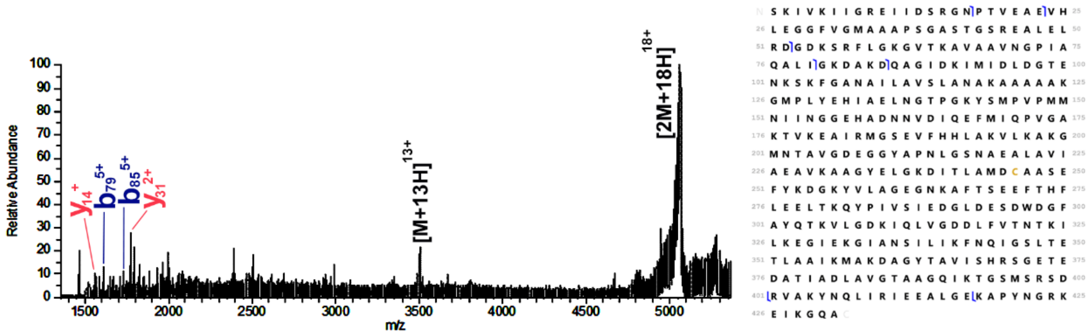

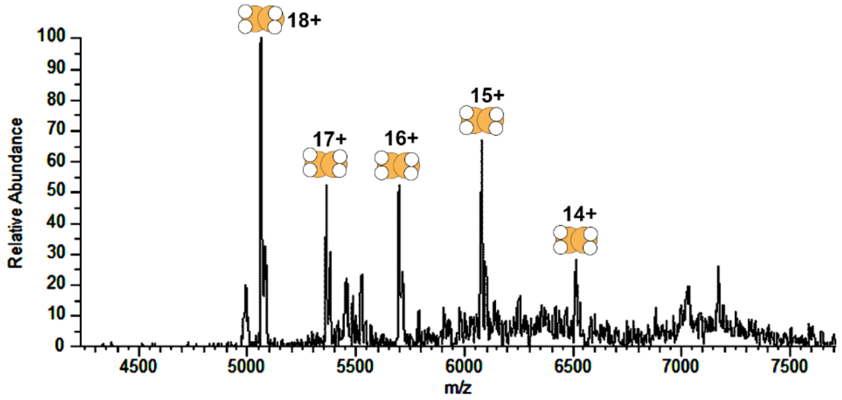

Name	m/z Monoisotopic	Mass Monoisotopic	Theoretical Mass	Error (Da)	Error (ppm)
b17	1880.1031	1879.0958	1879.1003	-0.0044	-2.3676
b23	2506.3823	2505.3750	2505.3914	-0.0164	-6.5439
b52	1791.5983	5371.7731	5371.7945	-0.0214	-3.9910
b79	1607.8670	8034.2988	8034.3227	-0.0240	-2.9846
b85	1730.7270	8648.5984	8648.6251	-0.0267	-3.0867
y14	1559.8651	1558.8578	1558.8579	-0.0002	-0.0988
y31	1771.9775	3541.9405	3541.9481	-0.0075	-2.1282

Section 2: Identification of proteins detected following (3+1) washing protocol

Table S2: Summary of proteins identified

Washing Protocol	Protein	m/z	Charge state	Protein mass calc (Da)	Protein mass observed (Da)	Mass difference (Da)	Sequence Coverage	Protein Stoichiometry Observed	Protein Type
3+1	Extracellular solute-binding protein family 3	3118	10+	31152.8914	31151.8444	-1.0470	2%	monomer	membrane related protein
3+1	Maltodextrin-binding protein	3700	11+	40681.9188	40681.9611	0.0423	1%	monomer	membrane related protein
3+1	Superoxide dismutase	3550<br>2550	13+<br>9+	45867.3212(dimer)<br>22934.6606(monomer)	45996.7417(dimer)<br>22934.4470(monomer)	129.4202(dimer)<br>-0.2136(monomer)	4%	homodimer	soluble protein
3+1	Phosphoenolpyruvate carboxykinase (ATP)	4217	14+	59606.0146	59930.0000	323.9854	1%	monomer	soluble protein
3+1	Cationic amino acid ABC transporter, periplasmic binding protein	4298	6+	25769.0150	25783.0000	13.985	3%	monomer	membrane related protein
3+1	Serine hydroxymethyltransferase	5066	18+	90577.9624(dimer)	91156.0000(dimer)	578.0376(dimer)	3%	homodimer	soluble protein
3+1	2,3-bisphosphoglycerate-dependent phosphoglycerate mutase	4406	13+	56815.4434(dimer)	56843.9280(dimer)	28.4846(dimer)	2%	homodimer	soluble protein
3+1	Long-chain fatty acid transport protein	5102	9+	45878.1115	45878.2669	0.1554	1%	monomer	membrane protein
3+1	Outer membrane protein A	4397	8+	35150.5051	35147.4616	-3.0435	1%	monomer	membrane protein

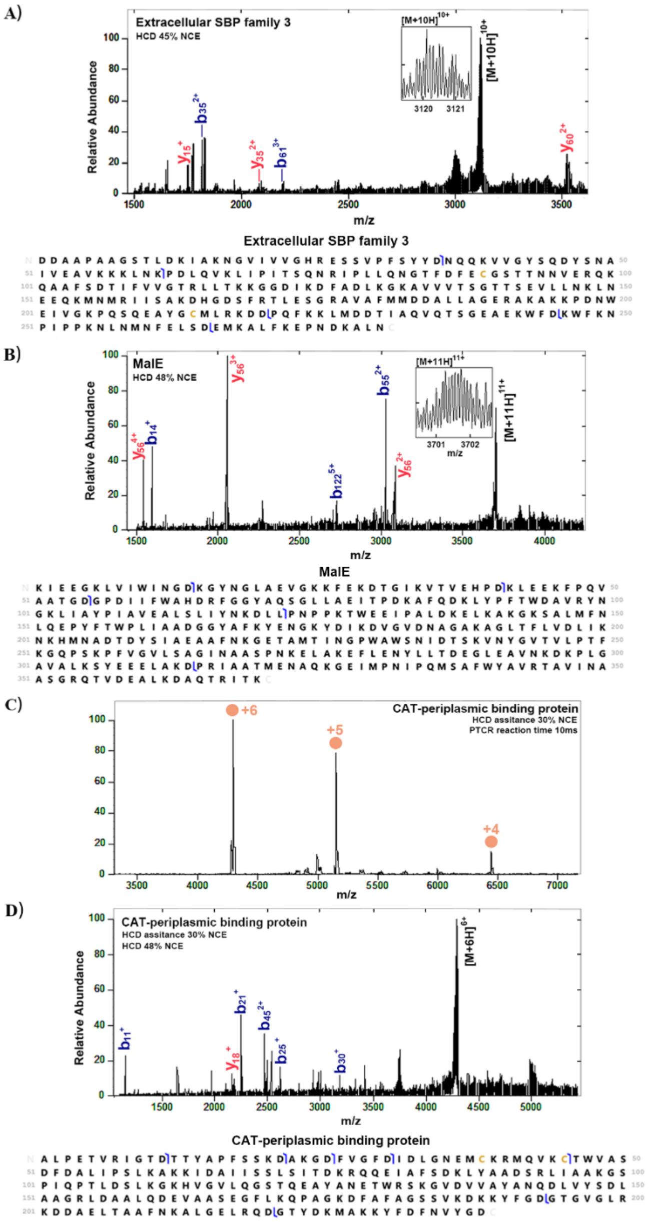

Figure S3. Identification of membrane associated proteins. **A)** HCD mass spectrum of 10+ ions (see inset) of extracellular SBP family 3 (m/z 3124±10), NCE 45% and sequence coverage. **B)** HCD mass spectrum of the 11+ ions (see inset) of MalE, NCE 48%, and sequence coverage. **C)** PTCR mass spectrum of and D) HCD mass spectrum (49% NCE) of 6+ ions of CAT-periplasmic binding protein precursor and sequence coverage.

Details of protein identification following washing protocol 3+1

1.Protein name: Extracellular solute-binding protein (SBP) family 3

Charge state: +10

Observed monoisotopic mass: 31151.8444 Da

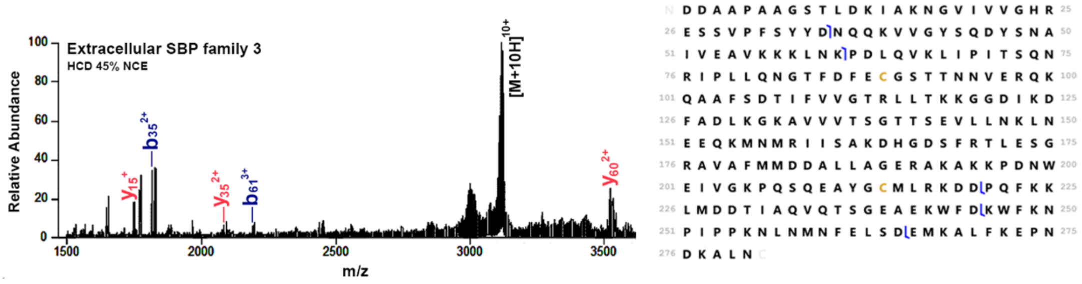

Name	m/z Monoisotopic	Mass Monoisotopic	Theoretical Mass	Error (Da)	Error (ppm)
b35	1816.4011	3630.7876	3630.7954	-0.0078	-2.1381
b61	2188.7990	6563.3752	6563.3724	0.0028	0.4263
b277	3084.6594	30836.5209	30836.7089	-0.1880	-6.0979
y15	1747.9087	1746.9014	1746.8974	0.0040	2.2898
y35	2081.0676	4160.1207	4160.1227	-0.0020	-0.4755
y60	3527.7643	7053.5141	7053.5183	-0.0041	-0.5848

2.Protein name: Maltodextrin-binding protein (MalE)

Charge state: +11

Observed monoisotopic mass: 40681.9611 Da

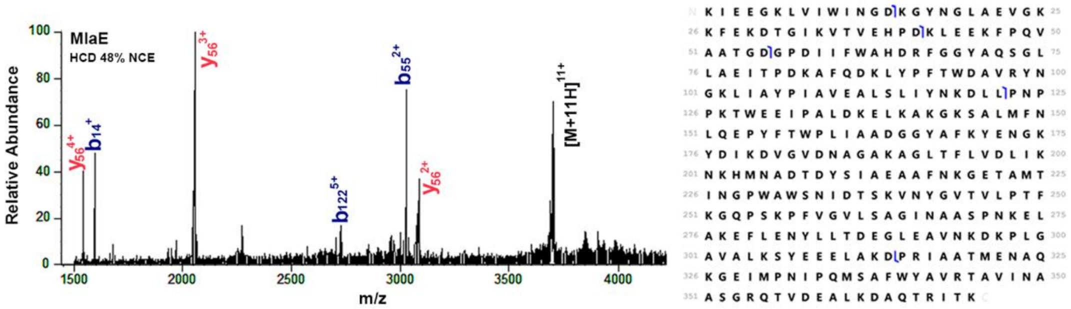

Name	m/z Monoisotopic	Mass Monoisotopic	Theoretical Mass	Error (Da)	Error (ppm)
b14	1595.8822	1594.8749	1594.8719	0.0031	1.9324
b41	2268.7037	4535.3928	4535.4063	-0.0135	-2.9686
b55	3025.5832	6049.1519	6049.1839	-0.0320	-5.2918
b122	2724.6179	13618.0530	13618.1119	-0.0589	-4.3276
y56	1540.5435	6158.1447	6158.1615	-0.0168	-2.7271
y56	2053.7234	6158.1484	6158.1615	-0.0131	-2.1251
y56	3080.0977	6158.1808	6158.1615	0.0193	3.1362

3.Protein name: Superoxide dismutase (SOD)
Charge state: +9, +13

Observed monoisotopic mass: 22934.4470(monomer without metal bond)
45996.7417(dimer with 1 Mn^{2+} binding each subunit)

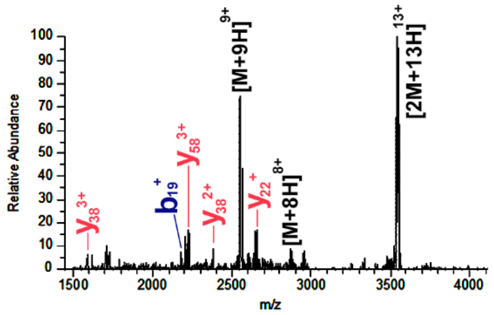

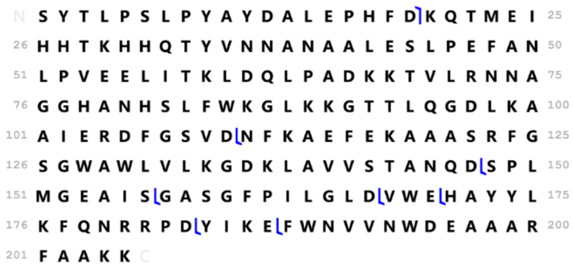

Name	m/z Monoisotopic	Mass Monoisotopic	Theoretical Mass	Error (Da)	Error (ppm)
b19	2180.9919	2179.9846	2180.0102	-0.0256	-11.7288
y18	2123.0582	2122.0510	2122.0748	-0.0238	-11.2315
y22	2656.3486	2655.3413	2655.3597	-0.0184	-6.9456
y35	2173.1069	4344.1993	4344.2133	-0.0140	-3.2160
y38	1587.1325	4758.3756	4758.4036	-0.0279	-5.8715
y38	2380.1962	4758.3779	4758.4036	-0.0257	-5.4022
y49	1929.6421	5785.9046	5785.9374	-0.0328	-5.6701
y58	2224.7719	6671.2940	6671.3640	-0.0700	-10.4899
y95	2667.0856	10664.3134	10664.4176	-0.1042	-9.7716

4. Protein name: Phosphoenolpyruvate carboxykinase (ATP)

Charge state: +14

Observed monoisotopic mass: 59930 Da

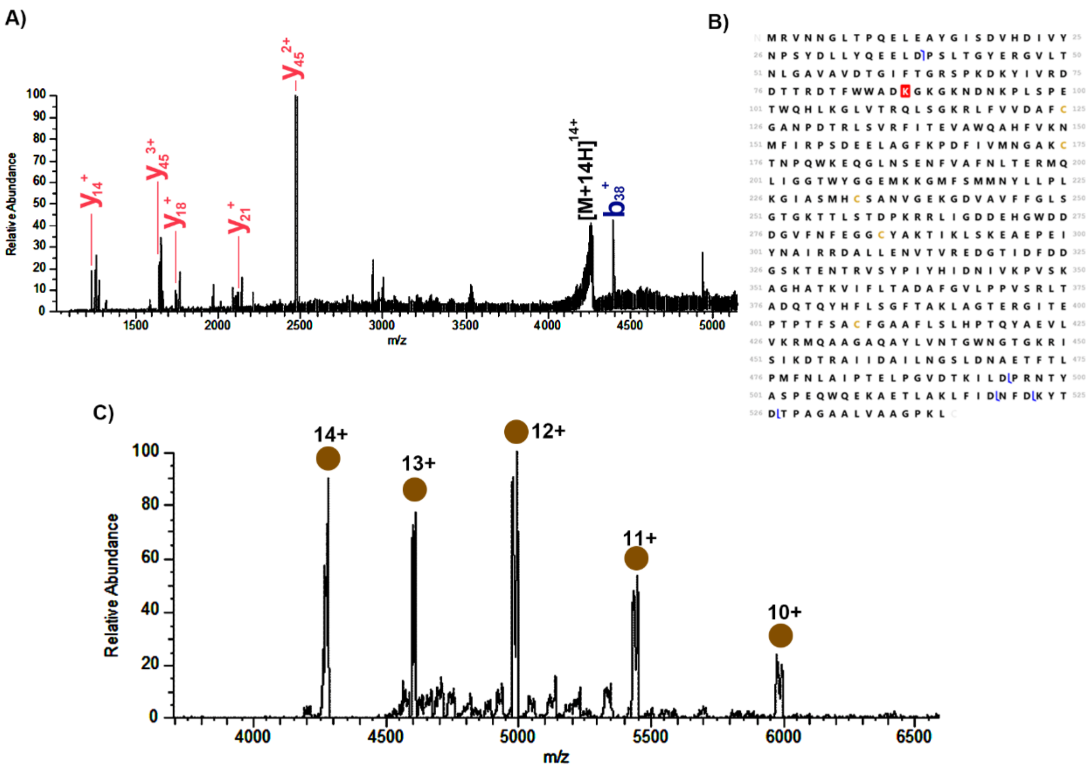

Name	m/z Monoisotopic Mass	Monoisotopic Theoretical Mass		Error (Da)	Error (ppm)
b38	4395.0618	4394.0545	4394.0688	-0.0143	-3.2630
y14	1236.7274	1235.7202	1235.7237	-0.0036	-2.8769
y18	1743.9605	1742.9532	1742.9566	-0.0034	-1.9582
y21	2120.0904	2119.0831	2119.0949	-0.0118	-5.5599
y45	2468.7459	4935.4772	4935.5082	-0.0310	-6.2826
y45	1646.1678	4935.4817	4935.5082	-0.0265	-5.3747

5.Protein name: Cationic amino acid ABC transporter, periplasmic binding protein

Charge state: +6

Observed monoisotopic mass: 25783 Da

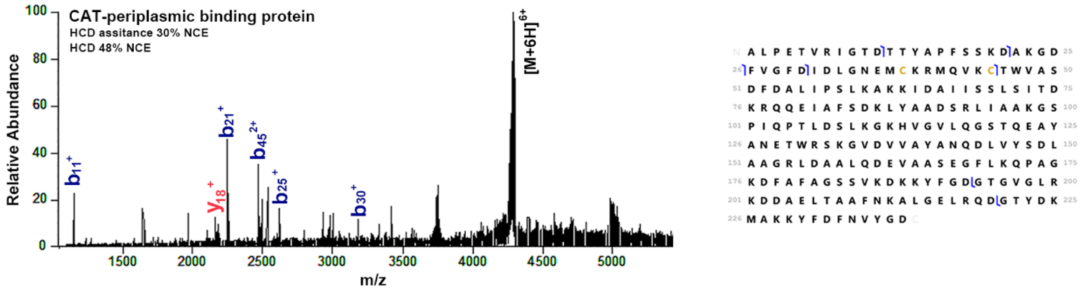

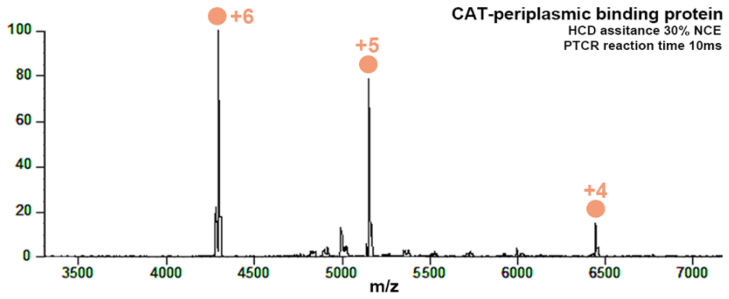

Name	m/z Monoisotopic	Mass Monoisotopic	Theoretical Mass	Error (Da)	Error (ppm)
b11	1153.6183	1152.6110	1152.6139	-0.0028	-2.4579
b21	2251.1052	2250.0979	2250.1168	-0.0189	-8.3911
b25	2622.2887	2621.2814	2621.2973	-0.0158	-6.0340
b30	3187.5429	3186.5356	3186.5509	-0.0153	-4.7873
b45	2468.7381	4935.4616	4935.3703	0.0913	18.4943
y18	2161.9747	2160.9674	2160.9826	-0.0152	-7.0213
y45	2494.7113	4987.4080	4987.4450	-0.0369	-7.4046

6.Protein name: Serine hydroxymethyltransferase

Charge state: +18

Observed monoisotopic mass: 91156 Da (Dimer)

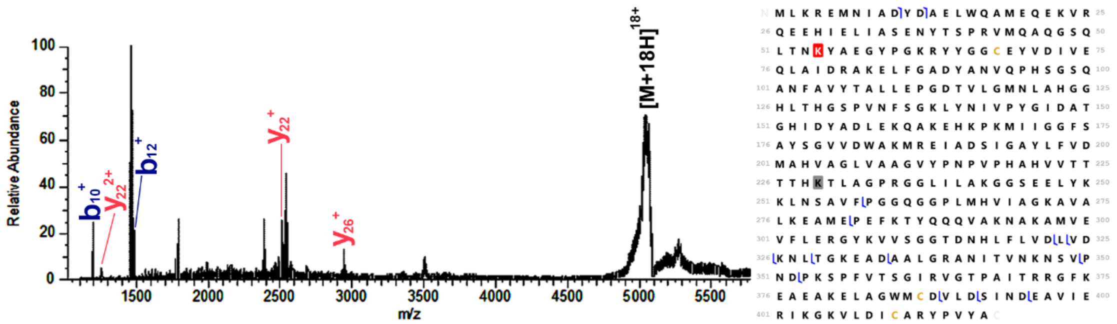

Name	m/z Monoisotopic	Mass Monoisotopic	Theoretical Mass	Error (Da)	Error (ppm)
b10	1202.5970	1201.5897	1201.5947	-0.0051	-4.2161
b12	1480.6857	1479.6784	1479.6850	-0.0066	-4.4658
y22	2506.3729	2505.3656	2505.3777	-0.0121	-4.8152
y22	1253.6909	2505.3672	2505.3777	-0.0105	-4.1734
y26	2935.5481	2934.5408	2934.5637	-0.0229	-7.7967
y26	1468.2780	2934.5414	2934.5637	-0.0223	-7.5923
y29	1631.8695	3261.7244	3261.7431	-0.0187	-5.7233
y65	1788.1832	7148.7036	7148.7405	-0.0369	-5.1603
y65	2383.9086	7148.7040	7148.7405	-0.0364	-5.0971
y68	1869.7118	7474.8180	7474.8631	-0.0450	-6.0263
y83	2246.9205	8983.6529	8983.7054	-0.0525	-5.8436
y83	1797.7381	8983.6542	8983.7054	-0.0512	-5.6976
y89	1917.9916	9584.9214	9584.9761	-0.0548	-5.7122
y92	2486.0415	9940.1369	9940.1981	-0.0612	-6.1554
y92	1989.0371	9940.1493	9940.1981	-0.0488	-4.9105
y94	2031.8536	10154.2316	10154.2934	-0.0618	-6.0868
y95	2054.4712	10267.3195	10267.3775	-0.0579	-5.6436
y136	2485.1177	14904.6623	14904.7515	-0.0892	-5.9854
y160	2156.8637	17246.8512	17246.9753	-0.1241	-7.1970
y160	2464.8466	17246.8751	17246.9753	-0.1002	-5.8083

7.Protein name: 2,3-bisphosphoglycerate-dependent phosphoglycerate mutase

Charge state: +13

Observed monoisotopic mass: 56843.9280 Da (Dimer)

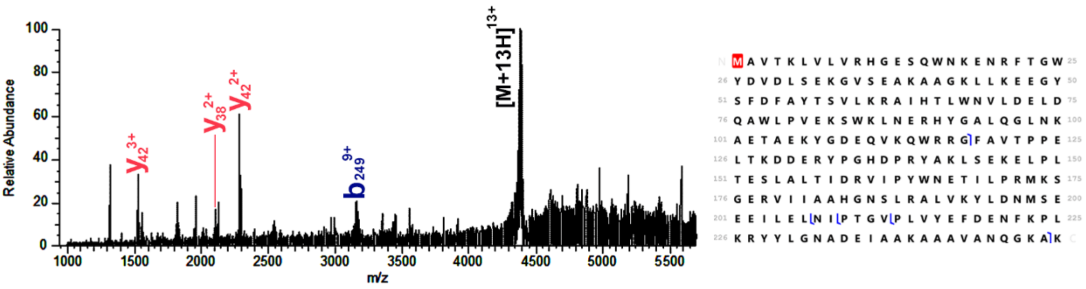

Name	m/z Monoisotopic	Mass Monoisotopic	Theoretical Mass	Error (Da)	Error (ppm)
b118	1958.5830	13703.0300	13702.9921	0.0380	2.7696
b249	3160.3843	28434.3935	28434.6595	-0.2659	-9.3521
y38	2107.0962	4212.1779	4212.2007	-0.0228	-5.4126
y42	2284.1861	4566.3577	4566.3910	-0.0333	-7.2874
y42	1523.1344	4566.3814	4566.3910	-0.0096	-2.1091
y44	2397.7550	4793.4954	4793.5180	-0.0225	-4.7030

8. Long-chain fatty acid transport protein

Charge state: +9

Observed monoisotopic mass: 45878.2669 Da

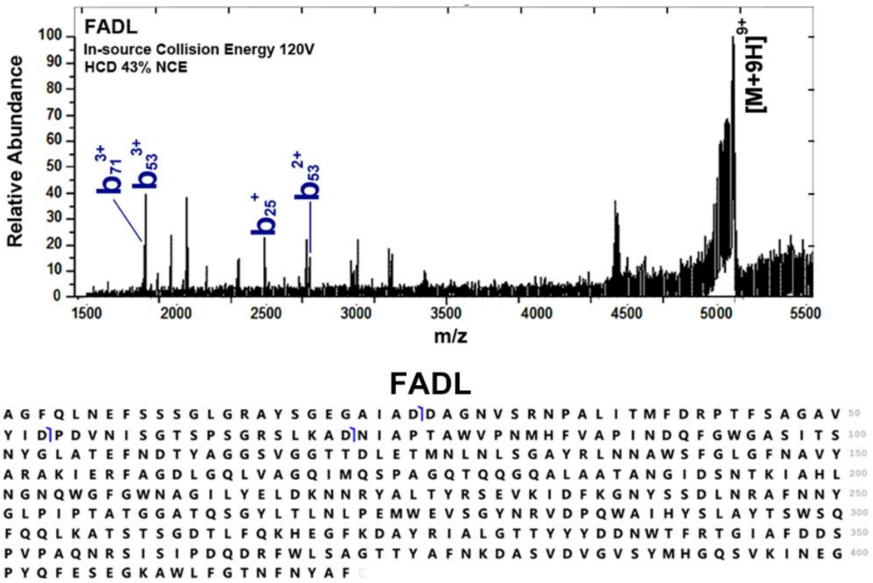

Name	m/z Monoisotopic	Mass Monoisotopic	Theoretical Mass	Error (Da)	Error (ppm)
b25	2486.1622	2485.1549	2485.1509	0.0040	1.6067
b53	2733.3053	5464.5960	5464.6058	-0.0098	-1.7881
b53	1822.5443	5464.6112	5464.6058	0.0054	0.9949
b71	1812.6254	7246.4727	7246.4965	-0.0239	-3.2918

9. Outer membrane protein A

Charge state: +8

Observed monoisotopic mass: 35147.4616 Da

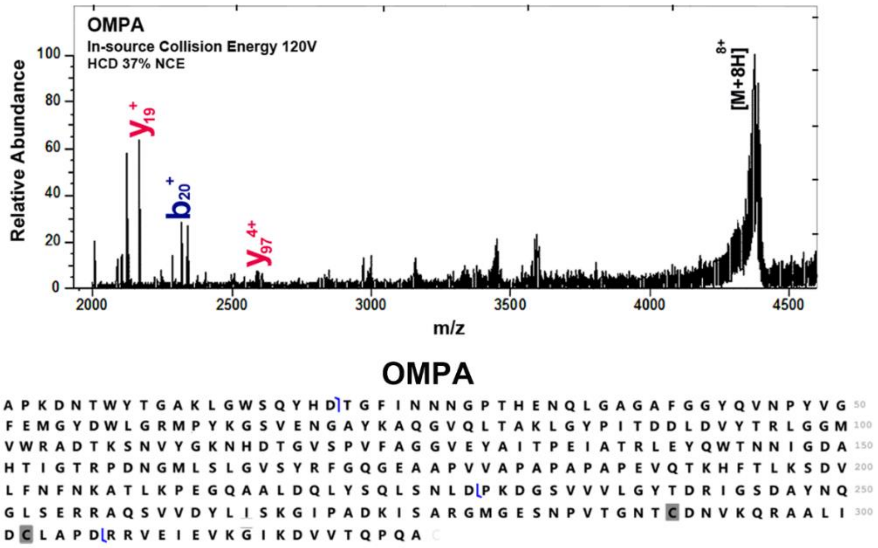

Name	m/z Monoisotopic	Mass Monoisotopic	Theoretical Mass	Error (Da)	Error (ppm)
b20	2320.0598	2319.0526	2319.0709	-0.0183	-7.8894
y19	2165.2386	2164.2313	2164.2328	-0.0015	-0.6797
y97	2595.5707	10378.2537	10378.3287	-0.0750	-7.2298
y264	3151.6124	28355.4463	28355.3784	0.0679	2.3959

References

[1] E.C. Randall, J. Bunch, H.J. Cooper, Direct Analysis of Intact Proteins from Escherichia coli Colonies by Liquid Extraction Surface Analysis Mass Spectrometry, Analytical Chemistry 86 (2014) 10504-10510.
[2] D.M. Horn, R.A. Zubarev, F.W. McLafferty, Automated reduction and interpretation of, Journal of the American Society for Mass Spectrometry 11 (2000) 320-332.
